# Post-operative endophthalmitis caused by *Acremonium falciforme* with orbital and extra-orbital involvement following combined cataract and glaucoma surgery: a case report

**DOI:** 10.1186/1752-1947-8-373

**Published:** 2014-11-19

**Authors:** Carlo Cagini, Alessia Iannone, Tito Fiore, Marco Lupidi, Leopoldo Spadea

**Affiliations:** 1 Department of Surgical and Biomedical Sciences, University of Perugia, Ospedale S Maria della Misericordia, Sant’Andrea delle Fratte, Piazza Menghini 1, 06156 Perugia, Italy; 2 Department of Biotechnology and Medical-Surgical Sciences, University of Rome “La Sapienza”, Via Benozzo Gozzoli 34, 00142 Roma, Italy

**Keywords:** Cataract surgery, Fungal endophthalmitis, Orbital involvement

## Abstract

**Introduction:**

In this report, we describe an unusual case of post-operative *Acremonium falciforme* endophthalmitis with orbital and extra-orbital involvement following combined cataract and glaucoma surgery.

**Case presentation:**

A 68-year-old Caucasian man with glaucoma presented with endophthalmitis characterized by pain, redness and impaired vision in the left eye fifteen days after combined cataract and filtering surgery. He subsequently underwent a pars plana vitrectomy, with vitreous sampling, silicone oil placement and intra-vitreal injection of antibiotics, but only after a second vitrectomy we identified *Acremonium falciforme* as the causative agent for the endophthalmitis. An antifungal systemic and topical therapy was started, but meanwhile the infection extended to orbital and peri-orbital tissues. Following these procedures, even if the eye went slowly in phthisis, we were able to limit the further extension and circumscribe the orbital and extra-orbital involvement.

**Conclusion:**

To our knowledge, this report is the first describe *Acremonium falciforme* endophthalmitis with orbital and extra-orbital involvement, following anterior segment combined surgery. Ophthalmologists and physicians should be aware of the extension risk of a fungal panophthalmitis, but also to potentially serious side effects related to systemic therapy.

## Introduction

Post-operative fungal endophthalmitis is a rare complication that frequently carries a worse prognosis than bacterial endophthalmitis. Approximately 90% of post-operative endophthalmitis cases develop after cataract surgery [1-5], and bacteria, such as coagulase-negative staphylococci and *Propionibacterium acnes*, are the most common causes. Various fungi are known to be major causes of delayed-onset endophthalmitis [6], and the prognosis appears to be related to many factors, including the extent of intra-ocular involvement, the timing and mode of intervention and the virulence of the organism involved. Although the outcome may be positive with vision recovery in some patients, in other cases the prognosis is not favorable [6]. Orbital and extra-orbital involvement is a rare complication of fungal endophthalmitis.

We describe a case of a patient with *Acremonium falciforme* endophthalmitis which developed two weeks after combined cataract and glaucoma surgery with important involvement of orbital and extra-orbital structures. To the best of our knowledge, there have been no other similar cases described in the literature to date.

## Case presentation

A 68-year-old Caucasian man with glaucoma who was receiving chronic therapy was referred to our institution with a diagnosis of post-operative endophthalmitis in the left eye. The patient had inflammation in the left eye that had started a few days earlier and was associated with impaired vision. Two weeks before presentation he had undergone cataract surgery combined with a glaucoma shunt implant at another eye clinic.

Upon presentation, he had a corrected distance visual acuity (CDVA) of 20/80 in the right eye, a cortical and nuclear cataract and filtering bleb that had developed after previous glaucoma surgery. Upon presentation, he had a corrected distance visual acuity (CDVA) of 20/80 in the right eye, a filtering bleb and a cortical and nuclear cataract developed after the previous glaucoma surgery performed two years earlier.

A glaucomatous optic disc excavation was present and the visual field was significantly narrowed in the inferior nasal area. He was receiving therapy with bimatoprost eyedrops in this eye.

His CDVA in the left eye was 1/200, and he showed marked conjunctival injection, corneal edema, inflammatory aqueous cells (Tyndall 3+) and fibrin on the front face of the intra-ocular lens (IOL). The glaucoma shunt implant (EX-PRESS® Glaucoma Filtration Device; Alcon Laboratories, Fort Worth, TX, USA) was in site with a non-inflamed filtering bleb. There was dense vitritis, and it was impossible to visualize the retina and the optic disc. A 25-gauge pars plana vitrectomy (PPV) was performed using a CONSTELLATION® vitrectomy system (Alcon Surgical, Tokyo, Japan), and infusion fluid (BSS PLUS®; Alcon Surgical) was devoid of any antibiotics. At a site 4.0mm from and parallel to the limbus, three trocars were inserted at a 30° angle (one in the inferonasal area for the infusion), creating tunnel sclerotomies. Initially, a vitreous sample (with the infusion channel closed) was collected using the vitrectome, then the remaining vitrectomy with hyaloid removal was completed. The vitreous cavity was filled with silicone oil, and ceftazidime and vancomycin were injected intravitreally. All the sclerotomies were sutured because of the risk of leakage.

**Figure 1:**
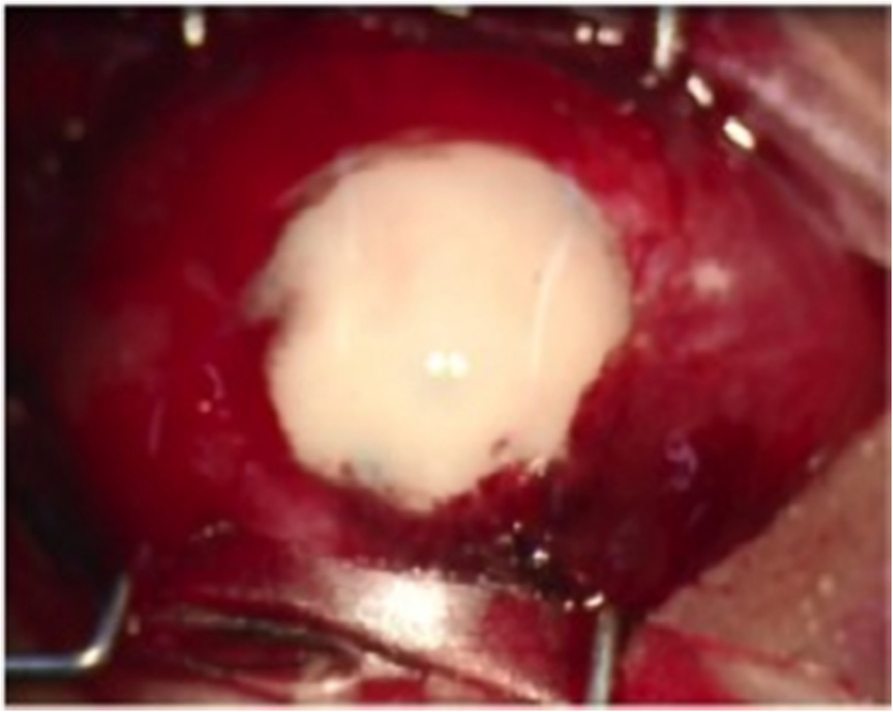
**Left eye of the patient before vitrectomy.** Pre-operative photograph shows diffuse subconjunctival hemorrhage and complete filling of the anterior chamber with hypopyon.

During surgery, the retina appeared to be covered by an abundant fibrinous exudation. After surgery, the patient’s CDVA was 1/30, and therapy with topical vancomycin (50mg/ml) and ceftazidime (50mg/ml) eyedrops six times per day was started. The IOL and the glaucoma valve were left in place because the patient strongly expressed this desire, given the low visual acuity of the other eye. Cultures taken from the aqueous and vitreous were negative, and his early post-operative course was uneventful and without signs of significant inflammation, except for a thin layer of fibrin on the front face of the IOL. He was discharged some five days after surgery, and he continued the therapy at home and returned for scheduled checks. During these checks, his CDVA was 1/20 and there were no signs of inflammation in the anterior chamber; however, the retina could not be easily evaluated, owing to fibrin plaque on the surface of the IOL.Forty-five days after this surgery, he experienced acute, increasing pain in the left eye with severe inflammation, corneal edema, anterior chamber inflammation (Tyndall 4+) and hypopyon occupying three-fourths of the anterior chamber (Figure 1). He underwent a new anterior chamber washout with aqueous sample and a vitrectomy with silicone oil tamponade.

**Figure 2:**
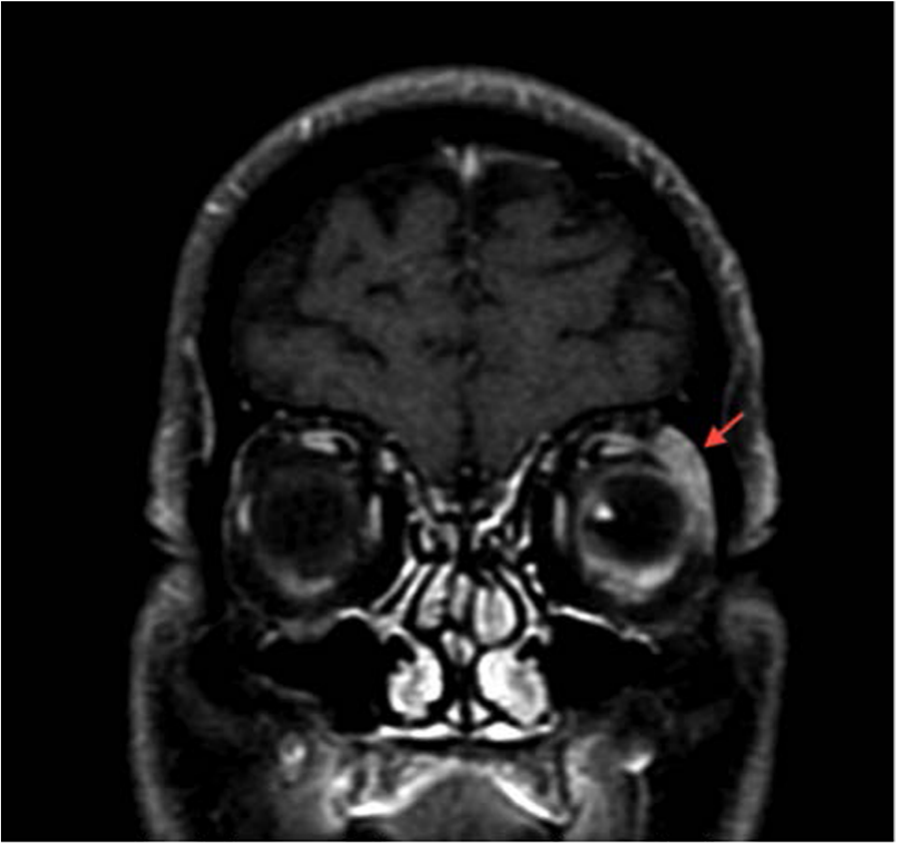
**Coronal T1-weighted magnetic resonance imaging scan.** Contrast agent was injected with fat suppression mode on. Peri-bulbar inflammation is more evident at the left lacrimal gland (red arrow).

**Figure 3:**
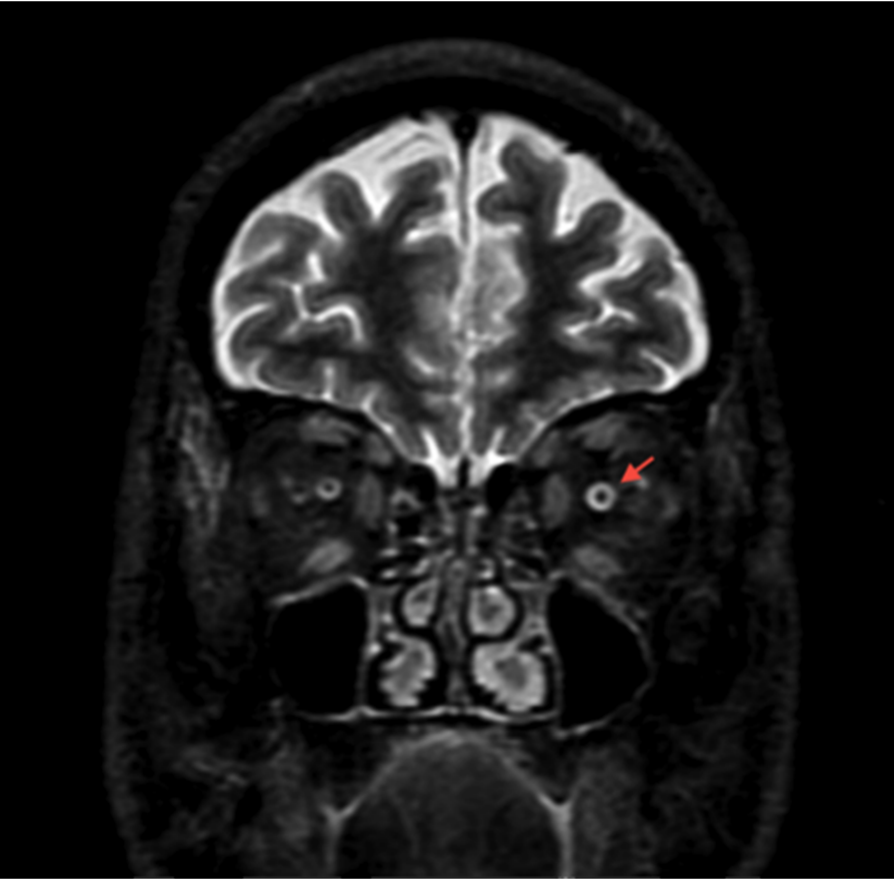
**Coronal short tau inversion recovery magnetic resonance imaging scan.** Faint hyperintensity of the left optic nerve can be seen in T2 (red arrow).

**Figure 4:**
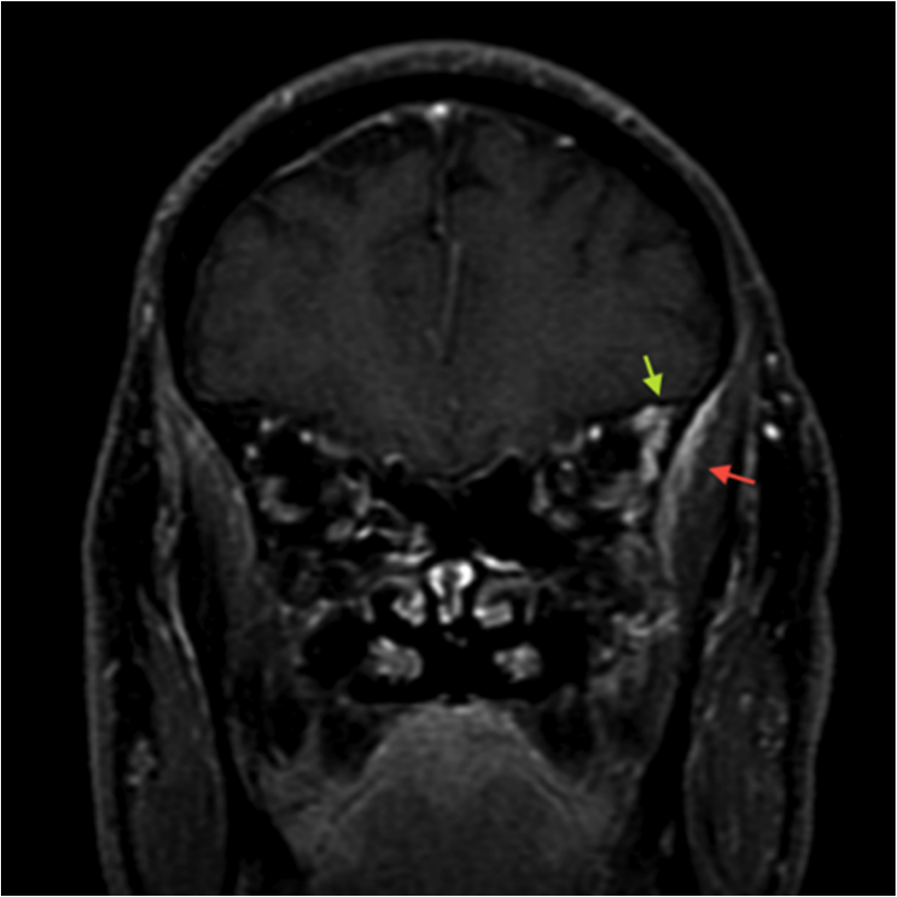
**Coronal contrast-enhanced, T1-weighted magnetic resonance imaging scan.** Contrast agent was injected with fat suppression mode on,, and enhancement of the left temporal muscle (red arrow) associated with ectasia of contiguous vessels (green arrow) is evident as an index of inflammation.

**Figure 5:**
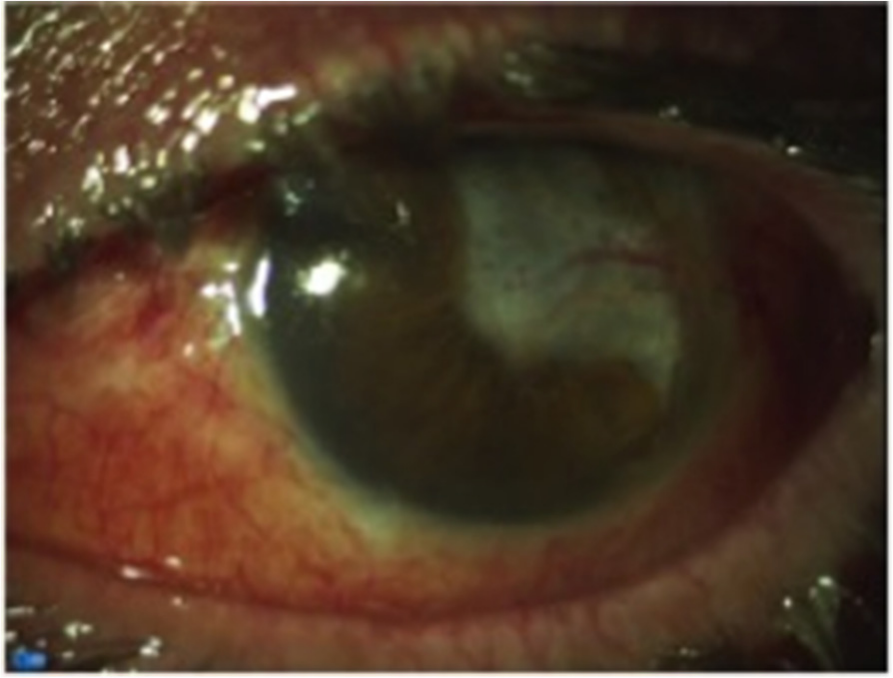
**Final appearance of the eyeball.** Photograph shows a wide corneal leukoma with limbal neovascularization involving the superotemporal area and pupillary region.

Two days after his second surgery, an increase of intra-ocular pressure was observed (treated with topical and systemic therapy) associated with the formation of a thick white plaque on the anterior surface of the IOL that prevented observation of the retina and the optic disc. His CDVA at this time was 1/200. Systemic therapy with fluconazole (400mg/day) and amphotericin B 0.15% eyedrops (six times per day) was started, which led to progressive reduction of the exudation in the anterior chamber. One week later, *Acremonium falciforme* species were identified in cultures obtained from samples, and the systemic therapy was switched to intravenous voriconazole 6mg/kg every 12 hours for the first day, then 4mg/kg intravenously every 12 hours for 10 days, followed by 200mg orally every 12 hours. This therapy led to a further reduction of the exudation in the anterior chamber. After seven days, it was possible to evaluate the red reflex of the retina, which revealed a slight improvement of CDVA to 1/60.Ten days later, the patient presented with liver function test impairment and sharp pain in the left orbital region that radiated to the same side of the head due to extension of the inflammatory process to the orbit and peri-orbital tissue. A magnetic resonance imaging (MRI) scan showed that there was evidence of left orbital inflammation with inhomogeneous appearance of the eyeball and peri-bulbar inflammation, which was more evident at the lacrimal gland (Figure 2). A faint hyperintensity of the optic nerve in T2 and slight signs of inflammation at the apex of the left orbital cavity were reported (Figure 3). At this time, therapy with systemic diclofenac and oral prednisone (25mg twice daily for two weeks) was started, which led to significant pain reduction. The condition of the left eye showed a gradual further improvement, but it was necessary to stop the systemic antifungal therapy after 22 days because of liver toxicity. The topical drugs were continued unchanged. One month later, unremitting pain in the orbital region reappeared; therefore, systemic antifungal therapy (voriconazole 6mg/kg intravenously every 12 hours for the first day, then 4mg/kg intravenously every 12 hours for 10 days, followed by 200mg orally every 12 hours) combined with pain therapy (acetaminophen 500mg/, codeine 30mg/day and gabapentin 300mg/day) was restarted, which led to progressive reduction of the patient’s ocular and peri-orbital pain.Immediately after this therapy, the patient showed significant ingravescent neurologic symptoms with visual hallucinations, postural instability, slight ideomotor slowdown, retropulsion and dynamic ataxia. Brain MRI excluded vascular lesions, but this examination highlighted enhancement of the left temporal muscle associated with ectasia of contiguous vessels, it was an evident index of inflammation (Figure 4). His electroencephalography results were normal, and lumbar puncture was performed to exclude infectious processes of the brain. Cerebrospinal fluid was clear, but showed albumin cytological dissociation. Once infectious or inflammatory processes were excluded, the cause of neurological symptoms was attributed to a toxic effect induced by the recently introduced analgesic therapy. After one week, we observed an improvement in neurologic symptomatology, but it was indispensable to maintain therapy with paracetamol, codeine and gabapentin. During this period, while the patient’s left eye began to show signs of phthisis, CDVA in his right eye decreased to 20/100 due to worsening of the cataract that was associated with progressive impairment of the optic nerve.After three months, voriconazole was suspended because of an increase in the cholestasis and hepatic cytolysis indices, but topical therapy was maintained for nine months. During this period, the patient was observed with close follow-up. He had no more signs of ocular inflammation; however, the white plaque on the anterior surface of the IOL did not disappear (Figure 5), and his pain was controlled with decreasing doses of analgesic oral therapy. The left eye’s condition deteriorated slowly into phthisis.

## Conclusions

In this report, we describe an unusual manifestation of post-operative *Acremonium falciforme* endophthalmitis with serious orbital and extra-orbital involvement in a fully immunocompetent patient. To our knowledge, *Acremonium falciforme* endophthalmitis after cataract surgery has been described only once previously [7] and no cases of *Acremonium* panophthalmitis have previously been reported in the literature.

Fungal intra-ocular infections sometimes show a specific pattern that can guide the specialist toward a prompt diagnosis. In other cases, they present an absolutely nonspecific manifestation and can be identified only by using one or more microbiological tests with aqueous or vitreous samples. This could be due to an uncertain medical history or, as in our case, to the advanced stage of inflammation at the time of the first visit. There are several reported cases of post-surgical, delayed-onset endophthalmitis. The wide majority of these cases are caused by bacteria. Only 0% to 4% of post-surgical endophthalmitis cases are caused by fungal infection. Therefore, after the negative outcome of the first cultures and in consideration of the severity of the clinical appearance in our patient, we established that a first therapeutic approach with broad-spectrum antibiotics associated with pars plana vitrectomy (PPV), is widely deemed the gold standard in fungal endophthalmitis management.

*Acremonium* species, formerly termed *Cephalosporium* species, are soil fungi that are ubiquitous environmental contaminants. They are saprophytic molds and have septate, colorless hyphae like those of other hyaline molds [8-10]. Although invasive disease may occur in an immunocompromised person, most cases of human disease, unlike other filamentous fungi, occur in immunocompetent hosts [11]. Ocular involvement of *Acremonium falciforme* is very uncommon.

Exogenous fungal endophthalmitis is known to occur in a variety of clinical settings, including contiguous spread of fungal keratitis, penetrating keratoplasty, cataract surgery, glaucoma filtering surgery, retinal detachment surgery and many others [9]. Moreover, *Acremonium* species may post-operatively invade through wounds, contaminated air solutions (such as humidifier fluid) or objects [10].

The initial symptoms of *Acremonium* endophthalmitis are similar to those of most delayed-onset endophthalmitis, including mild pain, redness, floaters and slightly decreased visual acuity [7-10]. The interval between surgery and endophthalmitis onset ranges from two to six weeks [12].

At present, no treatment modality for these fungal infections has been well established. Weissgold *et al*. [9] suggested that higher or repeated drug doses of amphotericin B (possibly in combination with vitrectomy) may be necessary to adequately treat these kinds of infections. Cameron *et al*. [7] reported that *Acremonium* remains viable in the anterior chamber despite surgical removal of the bulk of the fungal mass and treatment with several antifungal medications, including topical natamycin, topical amphotericin B, subconjunctival miconazole injection and oral ketoconazole. Joe *et al*. [13] treated the remaining white plaque in the anterior chamber after vitrectomy with voriconazole medication for six months. Mattei *et al.*[14] reported that voriconazole treatment appeared to be very effective in their case report of fungemia caused by *Acremonium*. Voriconazole is a triazole derivative that achieves a therapeutic level in aqueous and vitreous liquids by oral administration [14,15].

The possibility that the fungus remains in the glaucoma shunt implant or on the IOL despite topical and systemic therapy is well-known, and all the implants must be removed to eradicate the infection. We did not perform such a surgical procedure in our patient, first because he refused and later because of his poor general condition. We were aware of the risk of using systemic steroids to treat fungal infections, but the infectologist arranged this therapy while the patient was already taking a systemic antifungal drug. Moreover, we were faced with a post-operative infection with a subsequent inflammatory process that at first was apparently limited to the eye and the intra-orbital structures. We were unable to deliver the systemic antifungal therapy as we wished because of the risk of liver toxicity. Despite this rare infection that spread to the orbital, peri-orbital and temporal tissues, as well as the adverse events induced by the drugs (that is, liver toxicity and neurological impairment caused by antifungal therapy and analgesic drugs, respectively), we were able to manage the inflammatory damage and its complications.

## Consent

Written informed consent was obtained from the patient for publication of this case report and any accompanying images. A copy of the written consent is available for review by the Editor-in-Chief of this journal.

## Abbreviations

CDVA: Corrected distance visual acuity; IOL: Intra-ocular lens; MRI: Magnetic resonance imaging; PPV: Pars plana vitrectomy.

## Competing interests

The authors declare that they have no competing interests.

## Authors’ contributions

CC and AI analyzed and interpreted the patient data regarding the ophthalmic disease. TF performed the eye surgery. ML analyzed and interpreted the patient data regarding the orbital and extra-orbital involvement and was a major contributor to the writing of the manuscript. LS participated in coordination of the report and helped to draft the manuscript. All authors read and approved the final manuscript.
